# Finding the Best Thresholds of FEV_1_ and Dyspnea to Predict 5-Year Survival in COPD Patients: The COCOMICS Study

**DOI:** 10.1371/journal.pone.0089866

**Published:** 2014-02-27

**Authors:** Pere Almagro, Pablo Martinez-Camblor, Joan B. Soriano, Jose M. Marin, Inmaculada Alfageme, Ciro Casanova, Cristobal Esteban, Juan J. Soler-Cataluña, Juan P. de-Torres, Bartolome R. Celli, Marc Miravitlles

**Affiliations:** 1 Acute Geriatric Care Unit, Internal Medicine Department, Hospital Universitari Mutua de Terrassa, Terrassa, Barcelona, Spain; 2 Asturias Biomedical Research Office, Asturias, Oviedo, Spain; 3 Fundacio de Investigacio Sanitaria de les Illes Balears, Hospital Universitari Son Espases, Balearic Islands, Spain; 4 Respiratory Department, Hospital Universitario Miguel Servet, Zaragoza, Spain; 5 Respiratory Department, Valme University Hospital, Seville, Spain; 6 Respiratory Department, Hospital Nuestra Señora de la Candelaria, Tenerife, Spain; 7 Respiratory Department, Hospital Galdakao-Usansolo, Bizkaia, Spain; 8 Respiratory Department, Hospital Arnau de Vilanova,Valencia, Spain; 9 Respiratory Department, Clínica Universidad de Navarra, Pamplona, Spain; 10 Pulmonary and Critical Care Medicine, Brigham and Women's Hospital, Harvard University, Boston, Massachusetts, United States of America; 11 Pneumology Department, Hospital Universitari Vall d'Hebron, CIBER de Enfermedades Respiratorias (CIBERES), Barcelona, Spain; University of California San Francisco, United States of America

## Abstract

**Background:**

FEV_1_ is universally used as a measure of severity in COPD. Current thresholds are based on expert opinion and not on evidence.

**Objectives:**

We aimed to identify the best FEV_1_ (% predicted) and dyspnea (mMRC) thresholds to predict 5-yr survival in COPD patients.

**Design and Methods:**

We conducted a patient-based pooled analysis of eleven COPD Spanish cohorts (COCOMICS). Survival analysis, ROC curves, and C-statistics were used to identify and compare the best FEV_1_ (%) and mMRC scale thresholds that predict 5-yr survival.

**Results:**

A total of 3,633 patients (93% men), totaling 15,878 person-yrs. were included, with a mean age 66.4±9.7, and predicted FEV_1_ of 53.8% (±19.4%). Overall 975 (28.1%) patients died at 5 years. The best thresholds that spirometrically split the COPD population were: mild ≥70%, moderate 56–69%, severe 36–55%, and very severe ≤35%. Survival at 5 years was 0.89 for patients with FEV_1_≥70 vs. 0.46 in patients with FEV_1_ ≤35% (H.R: 6; 95% C.I.: 4.69–7.74). The new classification predicts mortality significantly better than dyspnea (mMRC) or FEV_1_ GOLD and BODE cutoffs (all p<0.001). Prognostic reliability is maintained at 1, 3, 5, and 10 years. In younger patients, survival was similar for FEV_1_ (%) values between 70% and 100%, whereas in the elderly the relationship between FEV_1_ (%) and mortality was inversely linear.

**Conclusions:**

The best thresholds for 5-yr survival were obtained stratifying FEV_1_ (%) by ≥70%, 56–69%, 36–55%, and ≤35%. These cutoffs significantly better predict mortality than mMRC or FEV_1_ (%) GOLD and BODE cutoffs.

## Introduction

According to the Global Burden of Disease Study, in 2010 chronic obstructive pulmonary disease (COPD) was the third leading cause of death worldwide and the ninth combining the years of life lost or lived with disability (DAILYs).[1], [2] COPD is characterized by an airflow limitation and therefore spirometry remains the essential test to diagnose the disease. Classically, COPD severity has been graded by postbronchodilator FEV_1_ expressed as percent of predicted values (FEV_1_ %).[3] More recently, several multidimensional indices have shown a better survival prediction than the isolated FEV_1_ (%). These include the original BODE index, which incorporates dyspnea measured with the modified scale of Medical Research Council (mMRC), Body Mass Index (BMI), FEV_1_, and exercise capacity assessed with the 6-minute walking distance (6MWD), as well as further modifications of this index, such as the BODEx (replacement of exercise capacity with severe exacerbations).[4]–[6] Other multidimensional prognostic indices are ADO (age, dyspnea, and FEV_1_), SAFE (quality of life measured by Saint George's Respiratory Questionnaire, FEV_1_, and 6MWD), and DOSE (dyspnea, smoking status, FEV_1_, and prior exacerbation history), among others.[7]–[9]

These indices have been constructed by adding different variables – such as dyspnea, exercise capacity, exacerbations, and age – to different categories of FEV_1_ values.[10] However, different thresholds of FEV_1_ and dyspnea are used in different staging systems and with different guidelines.[11]–[12] To date, the most widely used cutoff values are those proposed by the Global Obstructive Lung Disease (GOLD) and the ATS/ERS guidelines (mild≥80%, moderate 50–79%, severe 30–49% and very severe <30%).[13] However, the BODE index uses the old ATS standards (≥65%, 50–64% 36–49% and ≤35%), while the DOSE index uses a different cutoff (>50%, 30–49% and <30%).[4], [9] To the best of our knowledge the majority of these classifications are selected arbitrarily, based on cut-offs selected by expert opinion, and it is not known which of them best discriminates among different levels of mortality risk. Additionally, there are few studies comparing the usefulness of FEV_1_ in different age groups and the influence of dyspnea on survival assessment.[14]–[16]

The aim of the present study was to identify the best thresholds for FEV_1_ (%) and dyspnea measured with the mMRC to predict 5-yr survival in COPD patients, divided by subsets of age, using a pooled-analysis of individual patient data from eleven Spanish COPD cohorts (The COllaborative COhorts to assess Multicomponent Indices of COPD in Spain-COCOMICS study).[10]

## Methods

### Ethics Statement

All participants gave their informed written consent to participate, and their respective ethics committees approved each study (Hospital Galdekao-Usarsolo, Navarra Clinic University Hospital, Requena Hospital, Universitary Hospital Mútua de Terrassa, Universitary Hospital of Valme and Universitary Hospital Miquel Servet).

### Study design

The COCOMICS study is a pooled-analysis of individual patient-data from eleven Spanish COPD patient cohorts. The methodology has been described in detail elsewhere.[10], [17] Briefly a common data set with age, gender, spirometry, comorbidity, previous severe exacerbations, and follow-up among other variables was provided by the principal investigator of each of the participating cohorts.[6], [18]–[26] Previous severe exacerbations were defined as those requiring emergency room visit with or without subsequent hospitalization during the previous year. All-cause mortality at 5 years was defined as the primary outcome. Postbronchodilator forced spirometry was performed according to the guidelines of the American Thoracic Society/European Respiratory Society consensus.[27] Dyspnea was assessed using mMRC dyspnea scale.[5] Comorbidities were quantified by means of the Charlson index, excluding COPD, without adjustment for age.[28] All cohorts were previously published although with different follow-up periods; the references of original articles are available in the Online Appendix. All data were quality controlled centrally, and a homogeneous template to translate all coding was applied.

### Statistical analysis

Qualitative variables were expressed as absolute and relative frequencies, while quantitative variables were summarized as mean and standard deviation in the case of symmetry, and median otherwise. Comparison among continuous variables was made using the robust means comparison Student-Welch test, under symmetry, and the non-parametric Mann-Whitney U test otherwise. The Fisher exact test was used in order to check independence among categorical variables. We focused all analyses on time to death for all causes. Standard Cox semi-parametric proportional hazard models, all of them stratified by cohort, were used to study time-to-death data.[29] This methodology does not impose parametric restrictions on how the continuous covariate and the studied outcome are associated. In addition, the goodness-of-fit quality of the considered models was measured using the area under the incidence/dynamic ROC curve [30], AUC. The R package risksetROC, freely available in the R CRAN (www.r-project.org), was used for developing the computations. All the comparisons between the curves were performed using the L_1_-measure (given two functions, *f* and *g*, the L_1_-measure is defined by L_1_(*f,g*)  =  *∫ |f(t)−g(t)|dt*). The general Bootstrap Algorithm (gBA) [31] was used in order to approximate the respective P-values. The gBA method allows developing complex hypotheses preserving the original data structure and without assuming any additional hypothesis (just the one considered null). Finally, optimal % FEV_1_ and dyspnea thresholds were computed for maximizing the AUC at five years follow-up. In a first stage, the threshold which leads to the Youden index was computed and then, on each one of the two resulting sub-populations and with the same criteria, a new threshold was computed. In all analyses, P-values below 0.05 were considered for statistical significance. The free software R.2.15 was used for developing the analysis.

## Results

A total of 3,633 subjects with COPD (93% men) were included in the analysis, totaling the experience of 15,878 person-years. The mean age was 66.4 (SD ±9.7). At study entry, smoking exposure was substantial (53.4±26.5 pack-years), with 71.0% former smokers, and 27.9% current smokers. Most participants had moderate to severe airflow limitation with a predicted FEV_1_ (%) of 53.8%±19.4%, and a Charlson index of 2.1±1.5. Patients over 65 had significantly more comorbidities measured with the Charlson index than young people (≤64 years) [1.83 (1.44) vs. 2.17 (1.60); p<0.0001].

The main characteristics of the population cohorts are presented in Table 1. On average women were younger (59.8±11.0 years vs. 66.9±9.5 years) and more frequently current smokers (43.3% vs. 26.8%) than men (both p<0.05). After 5 years, 975 (28.1%) subjects had died. A significantly greater mortality was observed in older patients and women. Lower levels of FEV_1_ (%) and BMI, greater dyspnea, poorer quality of life measured with the Saint George's Respiratory Questionnaire (SGRQ), shorter distance walked in 6 x′ walking-test, and severe exacerbations of COPD during the previous year were also associated with a statistically significant five-year increased mortality (Table 1).

**Table 1:** Demographic and clinical characteristics.

Number (SD) o %	TotalN = 3,633	AliveN = 1,133	DeadN = 975	P	HR	CI 95%
Age	66.4±9.7	64.0±9.3	70.8±8.7	<0.001	1.07	1.06–1.08
Gender (men)	3,389 (93.3)	1,040 (91.8)	953 (97.7)	<0.001	2.99	2.10–4.24
SmokingYesNonFormer	26.9%1.2%71.9%	32.2%0.4%67.4%	20.8%2.1%77.1%	<0.0010.001<0.001	0.702.161 (Ref) &	(0.61–0.80)(1.43–3.27)–
SmokingPacks/year	53.4±26.5	55.4±27.3	56.6±26.8	0.101	1.01	(1.00–1.08)
Charlson Index	2.1±1.56	2.00±1.38	2.47±1.87	<0.001	1.18	(1.14–1.23)
FVC (%) predicted	80.2±22.6	88.6±22.4	69.0±20.7	<0.001	0.97	(0.97–0.98)
FEV1 (%) predicted	53.8±19.4	58.4±20.6	44.6±16.5	<0.001	0.97	(0.96–0.97)
FEV1/FVC ratio	51.9±11.8	51.3±11.9	48.6±12.0	<0.001	0.97	(0.97–0.98)
Dyspnea (mMRC)01234	17.6%33.7%27.8%23.7%7.2%	18.8%30.3%31.4%11.9%7.6%	10.0%24.4%29.8%21.5%14.3%	<0.0010.0070.495<0.001<0.001	1 (Ref) &1.241.792.683.25	–(0.99–1.55)(1.44–2.23)(2.13–3.39)(2.52–4.20)
Dyspnea (mMRC) #	1.59±1.14	1.03±1.20	2.03±1.20	<0.001	1.47	(1.40–1.54)
SGRQ*	42.7±20.4	43.1±20.2	47.6±19.2	0.003	1.01	(1.01–1.02)
BMI (Kg/m^2^)	27.9±4.98	27.7±4.82	27.1±4.99	0.018	0.97	(0.96–0.98)
6-minute walking test (meters)	397. 2±130.5	409.2±134.1	330.6±124.4	<0.001	0.996	(0.995–0.996)
Previous severe exacerbations ¥	0.89±1.82	0.91±2.22	1.28±1.86	<0.001	1.07	(1.05–1.09)

Five-year mortality.

& Reference group;

# mMRC: dyspnea measured with the modified Medical Research Council;

* Health related Quality of life, measured with Saint George's Respiratory Questionnaire;

¥ Number of hospitalizations for COPD exacerbation in the previous year.

**Figure 1:**
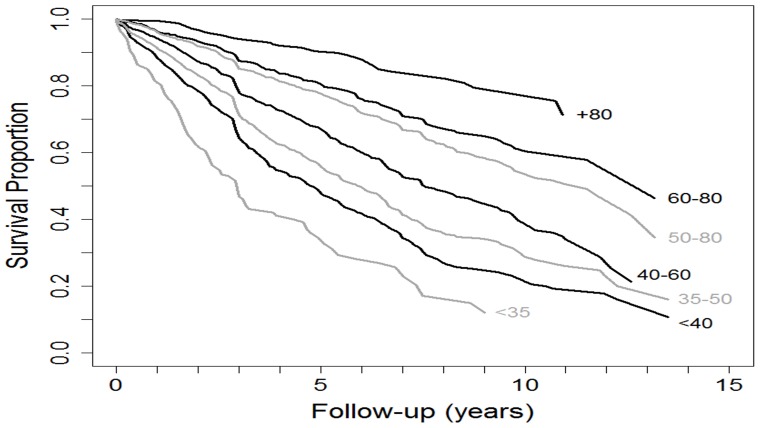
Kaplan-Meier survival curves for different thresholds of FEV_1_ up to 15 years. Hazard ratios and 95% confidence intervals for different thresholds of FEV_1_ :1) >80% reference group, 2) 60–79% (1.5; 1.06–2.11), 3) 50–79% (1.74; 1.25–2.43), 3) 41–59% (2.38; 1.7–3.32),4) 35–49% (2.92; 2.1–4.07),5)<40% (3.54; 2.53–4.95), 6)<35% (5.18; 3.53–7.61)

**Figure 2:**
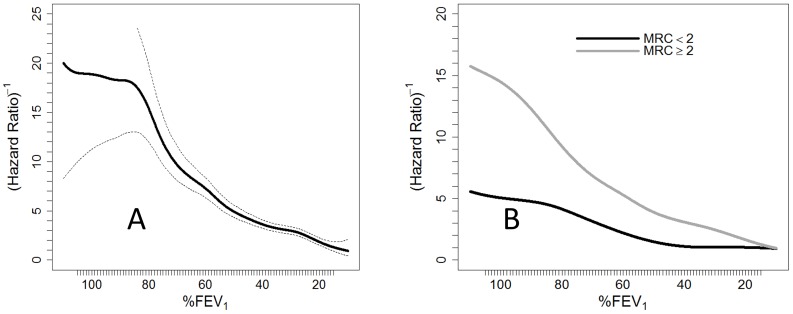
Spline inverse of the 5-yr hazard ratio of death to identify spirometry thresholds of severity (70%–55%–35%).

Mortality during short, medium and long-term follow-up – from one to 10 years - was consistently associated with FEV_1_ (%) (Figure 1, Table 2). The best FEV_1_ thresholds (%) in the entire cohort to predict 5-year mortality were mild≥70%, moderate 56–69%, severe 35–55%, and very severe ≤35%. Figure 2 shows graphically the risk of mortality at the different cutoffs of FEV_1_ (%). Of note, patients with an FEV_1_ (%) lower than 35% had a hazard ratio (HR) for mortality that was 6 times higher than the reference group with FEV_1_≥75% [95% Confidence Interval (CI): 4.69–7.74].

**Table 2:** Area under the curve (AUC) to predict 1, 3, 5, and 10-yr survival at different staging spirometry thresholds, dyspnea levels (mMRC) and time.

YEARS	1	3	5	10
COCOMICS	0.643	0.650	0.657	0.654
GOLD	0.635	0.639	0.647	0.639
Old ATS (BODE)	0.643	0.650	0.653	0.649
mMRC (Dyspnea)	0.623	0.620	0.625	0.614
P	0.013	0.006	0.004	0.006

GOLD: Global Obstructive Lung Disease classification. ATS: American Thoracic Society classification. BODE: Body Mass Index, Obstruction (measured with old ATS classification), Dyspnea and Exercise. mMRC: Dyspnea measured with the modified Medical Research Council scale.

**Figure 3:**
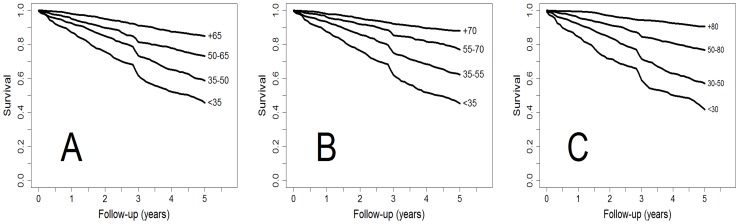
Kaplan-Meier survival curves of time to death by different staging spirometry thresholds: A) new COCOMICS, B) GOLD, and C) old-ATS/BODE.

The probability of survival at 5 years was 0.89 (95% C.I.:0.86–0.92) in patients with higher levels of FEV_1_ (%) (>70%) in contrast to 0.46 (95% C.I.:0.42–0.51) for patients with FEV_1_ (%) <35%. Kaplan-Meier curves for different thresholds of FEV_1_ (%) and 5-year mortality in the present study compared with GOLD and old ATS-BODE cutoffs are displayed in Figure 3.

For all comparisons, the predictive ability of the new cutoff points was higher than that of the previous cutoffs used in the GOLD document and slightly better than those used in the BODE index (old ATS). Hazard Ratios and their 95%CI between the different cutoffs of the COCOMIX study, GOLD, and BODE are presented in Table 3. Prognostic reliability of new thresholds is maintained at 1, 3, 5, and 10 years (Table 2).

**Table 3:** 5-yr hazard ratios of death at different staging spirometry thresholds.

	Mild	Moderate	Severe	Very severe	P
GOLD	>80%1 (ref.)	51–80%2.51 (1.83–3.44)	30–49%5.06 (3.71–6.90)	<30%7.83 (5.65–10.87)	<0.001
Old ATS (BODE)	≥65%1 (ref.)	50–64%1.95 (1.61–2.35)	36–49%3.02 (2.53–3.62)	≤35%4.62 (3.87–5.53)	<0.001
COCOMIX	≥70%1 (ref.)	56–69%1.91 (1.51–2.41)	35–55%3.40 (2.76–4.20)	≤35%5.57 (4.48–6.91)	<0.001

GOLD: Global Obstructive Lung Disease classification. ATS: American Thoracic Society classification. BODE: Body Mass Index, Obstruction (measured with old ATS classification), Dyspnea and Exercise. Hazard Ratios and 95% Confidence Intervals between different cutoffs of the COCOMIX study, GOLD, and BODE classifications.

The prognostic value of FEV_1_ (%) differed according to age. In patients 65 or older, we observed an inverse progressive relationship between mortality and lung function, while in individuals younger than 64, mortality was similar in the interval of FEV1% between 75% and 100%.

**Figure 4:**
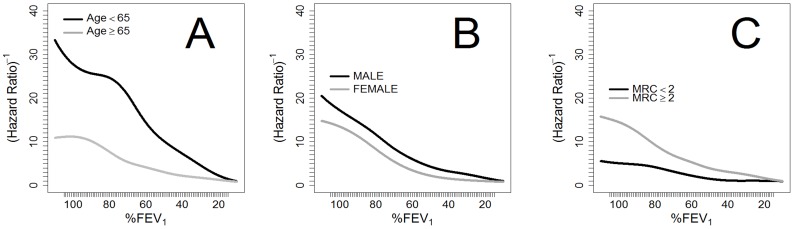
Spline inverse of the 5-yr hazard ratio for FEV_1_ and death, adjusted for: A) age, B) gender and C) dyspnea.

Among patients with lower levels of dyspnea (mMRC≤1), overall survival at 5 years was 75.6% (95% CI: 73.2–78.1), for those who also had lower dyspnea an FEV_1_ (%) >90% survival at 5 years was 92.1% (95% CI: 86.5–98.1). In contrast, patients with higher levels of dyspnea (mMRC≥2) had a 5-year survival of 56.0% (95% CI: 53.3–58.9). No differences existed in the predictive ability of FEV_1_ (%) by gender. The relationship between FEV_1_ (%), age, gender and dyspnea are graphically displayed in Figure 4.

**Figure 5:**
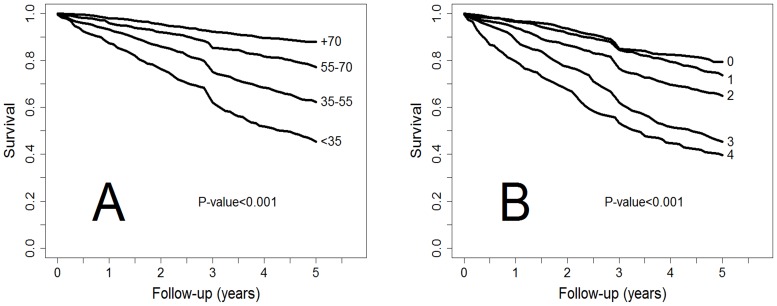
Kaplan-Meier survival curves up to five years for different thresholds of A) FEV_1_ according to new COCOMICS and B) mMRC. The quality of the models is measured from the AUC in the incidence/dynamic ROC curves.FEV_1_ is a better predictor of 5-year survival (p<0.005).

Of note, comparisons between Kaplan-Meier curves for the different levels of FEV_1_ (%) and dyspnea (Figure 5) showed that FEV_1_ (%) was a significantly better predictor of survival than degree of dyspnea (p<0.001). Similarly, the new cutoffs of FEV_1_ (%) were significantly better predictors of survival at 1, 3, 5, and 10 years than the levels of dyspnea measured with the mMRC (Table 3) (Figure 5).

## Discussion

The current study was conducted in a large sample of patients over the entire spectrum of COPD severity with long-term follow-up, and it shows that the proposed new spirometric thresholds to predict 5-year mortality (mild≥70%, moderate 56–69%, severe 35–55%, and very severe ≤35%) were significantly better predictors of survival than those used in the GOLD, and slightly better than those used in the BODE index (old ATS). This improvement in predictive capacity was also verified in both the short- and long-term follow-up (1 to 10 years). The study design – a pooled-analysis of individual patient-data from several cohorts – the large sample size and the different degrees of severity of the patients in the different cohorts guarantee a high external validity of the results.

Traditionally, lung function, measured with the FEV_1_ after a bronchodilator test, has been the most widely recognized variable associated with mortality in COPD. Furthermore, FEV_1_ is a good predictor of mortality even in the general population, and it is also considered the most important variable to evaluate the severity of COPD.[32] In addition, the decline in FEV_1_ over time has been used to evaluate the progression of the disease, although wide individual variability exist.[33] Until the last decade, different scientific societies and clinical guidelines had proposed different thresholds of postbronchodilator FEV_1_ expressed as percentage of predicted values to classify the severity of the disease. However these cutoffs were selected for pragmatic and educational reasons based on expert opinion, which explains the existing discrepancies in the proposed values.[3], [11] To the best of our knowledge, the present study is the first in which the different recommended thresholds were obtained from a cohort study looking for improved sensitivity and specificity points validated for mortality.

One important observation of our study is that although patients with lower levels of FEV_1_ (<35%) had a mortality that was 6 times higher than the patients with better FEV_1_ (≥75%), 5-year survival of patients with worse FEV_1_ (%) is almost 50%. In other words, higher values of FEV_1_ are associated with lower mortality, but a low FEV_1_, even below 35% predicted, does not exclude prolonged survival. These data are consistent with previous studies and highlight the conclusion that isolated FEV_1_ should not be used as an exclusive predictor of prognosis.[23], [34]

Accordingly, the new multicomponent indices have shown better predictive capacity for survival than FEV_1_ alone. The two common variables included in all multidimensional prognostic indices in COPD are respiratory function – measured with postbronchodilator FEV_1_ (%)– and dyspnea.[4], [6], [7], [9] However the cutoffs used are different among them. BODE index uses the old ATS values (≥65%, 50–64% 36–49%, and ≤35%), while the DOSE index uses a different cutoff (≥50%, 30–49%, and <30%), and the updated ADO index a 5 point scale (≥81%, ≥65–80, ≥50–64, ≥36–49 and ≤35%).[35] In contrast, the new GOLD document preserves its previous cutoffs for FEV_1_ (mild≥80%, moderate 50–79%, severe 30–49%, and very severe <30%), together with a combined COPD risk assessment evaluated with previous spirometric classification divided into 2 groups (FEV_1_%≥50% and ≤49%) along with the individual patient history of exacerbations in the preceding year (0–1 and ≥2 or 1 severe exacerbation). The evaluation of symptoms is measured with the COPD Assessment Test (CAT) (CAT<10 and CAT≥10) or dyspnea assessed by the mMRC scale (0–1 and ≥2), although the classification of COPD produced by the mMRC and CAT scores may differ.[3], [29] Additionally, several groups have demonstrated that the new GOLD multidimensional system classification does not improve prognostic reliability compared with the previous classification based only on spirometric severity for the prediction of mortality and hospitalizations. Indeed, mortality at 3 years was higher in GOLD group B (more symptoms, less risk) than in group C (more risk, fewer symptoms).[17], [36]–[38] A possible explanation is that patients in group B have more comorbidities, and therefore more symptoms despite better spirometric values.[39]–[40]

In the present study, the best cutoff for mMRC dyspnea scale was the same as that proposed in GOLD multidimensional system (mMRC≤1 or ≥2), and this confirmed that dyspnea is an excellent prognostic predictor of survival. In patients with lower levels of dyspnea, overall survival at 5 years was 75.6% while patients with higher levels of dyspnea survival decreased to 56.0%. However our study contradicts the findings reported by Nishimura et al. who reported that dyspnea is a better predictor of 5-year survival than airway obstruction in patients with COPD.[15] The most plausible reason for this discrepancy is that Nishimura's study was based on only 183 patients with severe impairment of FEV_1_ (mean 41%), while COCOMICS includes many more patients with a wider range of FEV_1_ values.

In our study, the predictive ability of FEV_1_ for 5-year mortality was similar between genders, but the relationship between FEV_1_ values and survival was different between patients younger than 65 years and those that were older. In younger patients, mortality was similar for values of FEV_1_ between 75% and 100%, drawing an initial curved plateau, with a progressively decreasing survival below these values. In elderly patients (≥65 years), we observed an inverse progressive relationship between mortality and lung function. Elderly patients also had more comorbidities and higher mortality during follow-up. The importance of comorbidities in COPD patients and their prognostic implications have been increasingly recognized in the last decade.[41]–[43] Heart disease, lung cancer, hypertension, musculoskeletal disorders, and diabetes, among many other diseases, are common in COPD patients, and several epidemiological studies have shown that lung function impairment is associated with an increased risk of comorbid diseases.[44] Previous studies highlighted how comorbidities were more closely related with mortality in older patients, while pulmonary function seemed to be more important in younger patients.[14] Several of these comorbidities affect spirometric values, diabetes and metabolic syndrome, and can lead to a somewhat restrictive pattern, with significantly lower FEV1 and FVC values than in non-diabetics, even after adjustment for age, sex, BMI, smoking status, diabetes duration, and HbA1c levels. Similarly heart failure, coronary artery disease, osteoporosis, hypertension, atrial fibrillation, and muscular or hormonal disorders are related with a reduced forced expiratory volume in spirometry.[45]–[48] However, all our patients met criteria for a pulmonary obstruction pattern, and decreased FEV_1_ is a recognized predictor of mortality not only in COPD but also in other diseases, and even in the general population.[49]

Our study has several strengths and limitations. Among the former are the large number of subjects included and the long follow-up period, including nearly 16,000 person-yrs. Both are essential to study mortality in a broad spectrum of COPD severity. Second, the follow-up information is very accurate, with few participants lost in follow-up. Third, all cohorts were recruited in Spain, and all investigators followed the same COPD clinical guidelines for pharmacological and non-pharmacological treatment.

However, several limitations need to be acknowledged. Firstly, the variables studied were measured at inclusion from each patient and our analyses assumed that the patients' condition did not change from baseline. It was not possible to make an analysis of time-dependent variables to assess changes in medication, smoking habits and other factors. Although some COPD variables show stability and repeatability, these analyses had no regular monitoring and re-staging.

Secondly, all our participants were Caucasian with a clear predominance of males, reflecting the epidemiology of COPD in Spain; therefore, our results should be extrapolated with caution in other populations.[50]

In conclusion our study performed in a large pooled-analysis of individual patient-data showed that the new spirometric thresholds (mild ≥70%, moderate 56–69%, severe 35–55%, and very severe ≤35%) predicted 5-year mortality significantly better than those used in the GOLD strategy and BODE index, and this improvement in predictive capacity was also verified in both short- and long-term follow-up (1 to 10 years).
